# Flexibility in Problem Solving: Analogical Transfer of Tool Use in Toddlers Is Immune to Delay

**DOI:** 10.3389/fpsyg.2020.573730

**Published:** 2020-10-06

**Authors:** Katarzyna Bobrowicz, Felicia Lindström, Marcus Lindblom Lovén, Elia Psouni

**Affiliations:** ^1^Department of Psychology, Lund University, Lund, Sweden; ^2^Public Higher Medical Professional School, Opole, Poland; ^3^Department of Philosophy and Cognitive Science, Lund University, Lund, Sweden

**Keywords:** analogical transfer, tool use, memory, toddler development, functionality

## Abstract

Solving problems that are perceptually dissimilar but require similar solutions is a key skill in everyday life. In adults, this ability, termed analogical transfer, draws on memories of relevant past experiences that partially overlap with the present task at hand. Thanks to this support from long-term memory, analogical transfer allows remarkable behavioral flexibility beyond immediate situations. However, little is known about the interaction between long-term memory and analogical transfer in development as, to date, they have been studied separately. Here, for the first time, effects of age and memory on analogical transfer were investigated in 2–4.5-olds in a simple tool-use setup. Children attempted to solve a puzzle box after training the correct solution on a different looking box, either right before the test or 24 h earlier. We found that children (*N* = 105) could transfer the solution regardless of the delay and a perceptual conflict introduced in the tool set. For children who failed to transfer (*N* = 54) and repeated the test without a perceptual conflict, the odds of success did not improve. Our findings suggest that training promoted the detection of functional similarities between boxes and, thereby, flexible transfer both in the short and the long term.

## Introduction

Adult humans solve problems continuously. As early as in toddlerhood, humans learn how tools allow for reaching goals that would otherwise be out of reach. This learning involves transferring solutions between problems, which, in turn, requires both an ability to identify those aspects of the problem that are relevant for the solution, and remember a solution long enough to be able to apply it again. While both these capacities allegedly begin to develop in infancy (Träuble and Pauen, 2007), they have – to date – only been studied separately from each other. The present study focuses on the joint contribution of these capacities at early stages of development of tool-dependent problem solving: a skill that underpins impressive human technological culture (Osiurak and Reynaud, 2019).

To discover which features of a tool are relevant for reaching a desired goal, infants attend to both the tool itself and its interactions with objects in the environment (Rakison and Woodward, 2008). In the first year of life, infants rapidly acquire knowledge about objects and interactions, both through own actions and through observing others (Leslie, 1984; Luo et al., 2009). In this process, perceptual features of the tool are linked to the effect that it exerts, that is, to the function it serves (Bates et al., 1980). This allows the infant to, by the end of the first year, shift from attending to overall perceptual similarity to attending to functional similarity when faced with unfamiliar objects, as long as the common function is demonstrated beforehand (Träuble and Pauen, 2007).

Thus, it seems that 11- and 12-month-olds not only acquire, but also transfer knowledge about the functional parts of objects; for instance, they can identify a toothbrush and a dish brush as sharing the same functional part after observing washing the dishes with the latter. However, at this age, if a shovel had a handle similar to that of a dish brush but different from that of the toothbrush, the child would most likely group the dish brush and the shovel as similar to each other, and as different from the toothbrush. In other words, 12-month-olds still fail to prioritize the object’s functional features over conflicting perceptual ones (Madole et al., 1993; Booth and Waxman, 2002). Resolving such conflicts may develop much later, as even 24-month-olds may struggle with disregarding the perceptually salient yet misleading features of a tool in favor of the functionally relevant ones (Baker and Keen, 2007; Bechtel et al., 2013; Pauen and Bechtel-Kuehne, 2016 but Chen et al., 1997). Although 24-month-olds still prioritize perceptual saliency over the tool’s function, contrary to younger infants, they rapidly improve their performance upon feedback. The improvement in selectively attending to the object’s function in the second year of life coincides with the onset of tool use in everyday life, e.g., eating with a spoon (Conolly and Dalgleish, 1989; McCarty et al., 2011; Bechtel et al., 2013).

Solving problems with tools poses a twofold difficulty: one must identify the functionally relevant features within the tool on the one hand, and the functionally relevant features within the problem on the other. For instance, to open a door, one needs to find not only the right key, but also the keyhole. Relying on such functional matches between problem and tool supports transferring solutions across problems and therefore boosts behavioral flexibility. The ability to detect common principles for solution across problems, termed analogical transfer, improves between the second and fourth year of life. For instance, Crisafi and Brown (1986) showed that 2-year-olds failed to transfer spontaneously across problems, several 3-year-olds transferred a solution across physically similar problems, and many 4-year-olds transferred across physically dissimilar problems that did not require tool use. Likewise, Brown and Kane (1988) showed that 3-year-olds spontaneously transferred tool knowledge to a target story but needed a slightly longer exposure to the source story compared to 4- and 5-year-olds. Other studies have corroborated this developmental trajectory (Holyoak et al., 1984; Brown, 1989; Brown et al., 1989; Goswami, 1991; Chen, 1996). Importantly, none of these studies required actual tool use.

Between the second and fourth year of life, children rapidly develop a skill that is a hallmark of everyday human life: flexible tool-dependent problem solving. Its flexibility in adults is boosted by well-developed long-term memory, which allows for transfer across both immediate and delayed situations (Bobrowicz, 2019), but the interaction between long-term memory and analogical transfer of tool use has been overlooked in research, regarding both adults and children. This is somewhat surprising, considering the role that analogical transfer of tool use has played in the development of human technological culture (Osiurak and Reynaud, 2019). Such development cannot rely solely on analogical transfer of tool use; it must be supported by long-term memory that allows transferring technical skills between dissimilar contexts. The present study focuses on the interaction between analogical transfer, tool use and long-term memory in development.

Long-term memory, including episodic and procedural memory, supports analogical transfer across situations that, in everyday life, are often separated by hours, days or even months. It is unclear when long-term memory begins to support analogical transfer in young children, but previous findings suggest that immaturity of long-term memory, especially episodic memory, may limit transfer between contexts before the age of 3. While procedural memory allows for issuing motor actions acquired in the past, episodic memory warrants flexible retrieval of relevant previous situations. While not much is known about the development of procedural memory between the second and fourth year of life, brain structures associated with procedural memory mature ahead of the structures associated with episodic memory. Thus, it is reasonable to assume that procedural memory matures earlier than episodic memory (Bauer, 2008) but findings are scarce and contradictory (Lum et al., 2010). In any case, it seems that episodic memory is available to 3-year-olds, but improves further between the ages of 3 to 5, particularly in terms of richness of information about past situations (Hayne et al., 2011), retention interval (from 15 min in 3-year-olds to 24 h or even a week in 4-year-olds; Scarf et al., 2013), and recollection of the temporal aspects of past situations (Scarf et al., 2017). What does not improve, however, is the accuracy of recollection (Hayne et al., 2011), the ability to form episodic memories (Scarf et al., 2013), and the recollection of “what” and “where” happened in the past situation (Scarf et al., 2017).

Findings from episodic memory research suggest that children younger than 4 years could struggle with applying knowledge to a target problem 24 h after training on a relevant source problem. However, previous studies with deferred imitation (Herbert and Hayne, 2000) and object search (DeLoache et al., 2004) tasks demonstrated that even 2.5-year-olds can use long-term memory to transfer knowledge across problems, both immediately after training on the source task and 24 h later. This suggests that even before the age of 3, children can identify and flexibly apply relevant knowledge acquired on another, functionally similar task.

In the current study, we investigated the development of flexible tool-dependent problem solving in children 2- to 4-years old, focusing on tool use and analogical transfer across immediate and delayed situations. We limited language demands of the task to a minimum so that language abilities would not influence the children’s performance. We designed a novel experimental setup that required solving a problem through physical tool use, after training on an analogical problem. A control group did not receive this training. Children carried out the test task either shortly after training, or after a 24-h delay. This manipulation allowed for investigating the impact of short-term and long-term memory on analogical transfer. Children who failed to solve the test task with the perceptually incongruent tool set were further tested with another tool set, where the perceptual mismatch had been removed. This manipulation, to a limited extent, allowed for investigating the impact of perceptual mismatch on analogical transfer.

We predicted that:

(H1) With age, children would be more likely to solve the test task after training, regardless of the perceptual mismatch within the tool set.

(H2) Compared to older children, younger children would be less likely to solve the test task after 24-h delay.

In order to investigate ability to focus on the aspects relevant for solving the problem, the setup involved a twofold difficulty – prioritizing the functionally relevant features of the problem over the irrelevant ones, and prioritizing the functionally relevant features of the tools over the irrelevant ones. Therefore, children needed to not only transfer a solution between two perceptually dissimilar problems, but also overcome a mismatch (conflict) between the perceptual and the functional features of the tools. We predicted that:

(H3) With age, children who received training would interact longer with the functional tool and the relevant components of the apparatuses; showing that they successfully identified the relevant aspects of the target problem. Thus, with age, children who received training will also interact shorter with the functional tool and the irrelevant components of the apparatuses.

(H4) Compared to older children, younger children receiving the test task with a 24-h delay would interact shorter with the functional tool and the relevant components, but longer with the irrelevant components, showing difficulties with identifying the relevant aspects of the target problem.

(H5) Children who solved the test task would interact longer with the functional tool and the relevant components of the apparatuses, compared to children who did not. While interacting with the functional tool and the relevant components is critical to solving the target problem, such interactions do not guarantee the solution if children do not know how to correctly apply the tool’s function.

(H6) Removing the perceptual mismatch may not benefit children who failed to solve the test with the perceptually mismatching tool set, since children between 2 and 4.5 years should be able to prioritize tool function over irrelevant perceptual similarities.

## Materials and Methods

### Participants

In total, 122 children were recruited from eleven public preschools in urban and semi-urban areas of southern Sweden. Focusing on the ages of 2 to 4 years, we aimed at children 21–51 months old, but seven children who were 52–55 months old were also included. The children’s average age was 40.42 months (SD = 7.49). Parental education was high, with 86.6% having a college or university degree. Most children (57.7%) had one sibling, 13.8% had no siblings, and 21.1% had two siblings or more. Only data from 105 children (51 boys/54 girls) were included in the present analysis. Data from 17 children were excluded because of missing or distorted video recordings (***n*** = 12), successful solution of all test tasks upon the first presentation (***n*** = 2, 43, and 48 months), or missing the second day of testing (***n*** = 3). Out of all children, 90 received training while 15 did not, serving as a control group. The control group was limited to 15 children as a trade-off, to increase the power of the statistical analyses conducted for the experimental group. The children’s mean age in the control group was 38.53 months (SD = 9.09).

This research was approved by Swedish Ethical Review Authority in Lund (DRN 2018/572, PI Psouni). No sensitive data about participants was gathered, and only those children, whose parents submitted a written consent, either on paper or digitally, were included in the study.

### Materials

**FIGURE 1:**
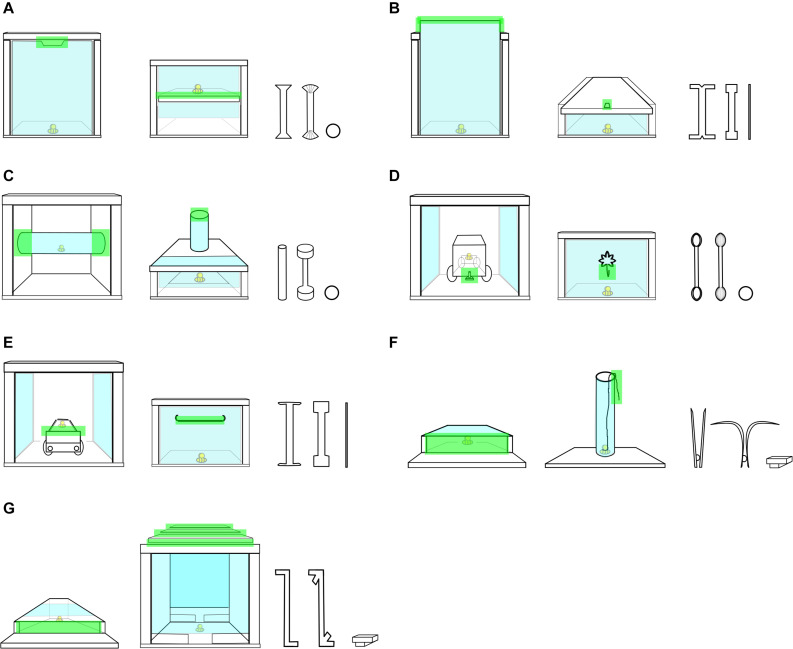
An overview of all sets of apparatuses and tools used in the study, with relevant components highlighted in green. Within each set of apparatuses, the training task is depicted to the left and the test task to the right. Within each set of tools, the functional tool is depicted to the left, the non-functional in the middle, and the useless to the right. Simple motor actions were required to open each box: **(A)** inserting the tip of the functional tool into the gap in the upper part of the apparatus (training) or the middle (test), and then lifting the tool’s handle; **(B)** hooking the tip of the functional tool onto the upper part of the door (training) or the hole in the front part of the lid (test), and then pulling the tool’s handle; **(C)** inserting the tip of the functional tool into the tube’s opening, and pushing the tool’s handle to the side (training) or downward (test); **(D)** casting the loop-like tip of the functional tool onto the car’s hook (training) or the hook on the doors (test), and pulling the tool’s handle; **(E)** casting the rake-like tip of the functional tool onto the car (training) or the handle on the doors (test), and pulling the tool’s handle; **(F)** inserting the tips of the pincette-like functional tool, grasping the bee, and pulling the tool (training) or using the tips to grasp the string protruding from the tube, and pulling the tool (test); **(G)** inserting the tip of the hockey-bat-like functional tool, and raking the bee out (both training and test).

Seven sets of tool-use tasks were developed, each with two puzzle boxes and three tools (see Figure 1). Each set consisted of a training task and a test task, which looked different but required a similar solution. The puzzle boxes were made of medium-density fibreboard (MDF) and each had a transparent plexiglass surface through which the child could peek at a toy bee trapped inside. The MDF was covered with non-toxic paint to make the boxes seem “dirty” and so discourage the children from touching them with bare hands and try to retrieve the bee using tools instead. The experimenter always wore gloves or used a paper towel to handle the boxes to make this story more believable, and children were playing along.

Each set of tasks was accompanied by a set of three tools: a functional, a non-functional and a useless one, all made of white FIMO clay. The functional and the non-functional tools had the same length and rigidity, but different ends. The functional tool had a functional element on both ends, and, paired with a correct motor action on the child’s part, allowed for retrieving the bee. The non-functional tool likewise had a non-functional element on both ends, so regardless of the motor action executed by the child, it did not allow for retrieving the bee. Finally, the useless tool differed in length, shape and rigidity from the other two and did not allow for solving the task (Figure 1). Within each set, solving both the training and the test task required the same tool and the same motor action (see Supplementary Figure S1). Each child was tested with one set.

Although the same set of tools was used in both the training and the test task, we manipulated the appearance of the tools. In the baseline and the training, each of the tools had a unique salient pattern painted on the white clay with a non-toxic dark-blue pen. The patterns on the functional and the non-functional tools were swapped in the test, leading to a perceptual mismatch between the baseline/training and the test.

**FIGURE 2:**
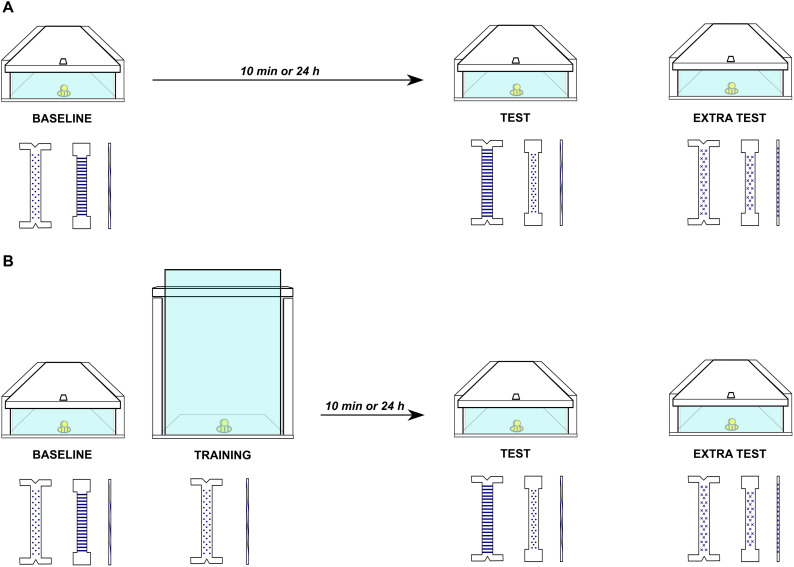
An overview of the procedure in the two experimental conditions: without training **(A)** or with training **(B)**. Each child was first presented with a puzzle box containing a toy bee visible behind a transparent surface and three tools. If the child could not open the box, s/he either waited 10 min or 24 h before another chance to open the box **(A)** or s/he received a training on another box that required the same solution **(B)**. After the training, she had another chance to open the original box, either right afterward or 24 h later. This figure shows the “**B**” set that required either hooking the tip of the functional tool onto the upper part of the door (training) or the hole in the front part of the lid (test) to solve the task.

In an extra testing round that commenced after a failed test, the perceptually mismatching set of tools was substituted with another set, comprising a functional, non-functional and useless tool, all decorated with a uniform x-pattern (see Figure 2).

### Procedure

Children were recruited through announcements at the preschools, after active, informed consent by their parents/guardians. Information about the study was both physically and digitally available to parents. Test leaders spent a day in each preschool to become acquainted with the children. Children were tested individually, at their preschools, at a room arranged for the purposes of the experiment. All trials were video-recorded, capturing the experimental setup and the participant’s hands. Before each trial, children were engaged in a short chat about the box being dirty and the bee trapped inside the box. If the children had not been interested in the task at this point, the experimenter would not have proceeded with the baseline. However, such problems did not occur, as all children were interested in the trapped bee and releasing it, interacting with tools and the apparatus.

The experiment employed a 2 × 2 factorial design, manipulating condition (training vs. no training) and delay (short/10 min vs. long/24 hrs). At baseline, the child received a single opportunity to interact with the test task, from picking up a tool, through using it on the apparatus, to abandoning the tool, to ensure that he/she could not solve the task spontaneously. All three tools were available: a dot-patterned functional, a stripe-patterned non-functional and a wave-patterned useless one. If the child chose the functional tool, used it in a correct way and released the bee, a test task from another set was presented.

Upon failure to solve the task at baseline, children were assigned into either training (experimental) or no training (control). Children in the experimental group began the training immediately after baseline. During training, two tools were available: a dot-patterned functional and a wave-patterned useless one. Now, the child learned, with the help of the experimenter, how to use the functional tool to retrieve the bee from the training task. The child was first encouraged verbally to try to release the “trapped” bee from the box. If the child did not succeed, the experimenter would demonstrate once how to use the functional tool on the relevant components of the task. The child did not receive any verbal instructions and, therefore, relied solely on the motor demonstration. After each demonstration, the child was verbally invited to try on his/her own. This sequence of demonstrations by the experimenter and attempts by the child were repeated until the child succeeded to retrieve the bee thrice without any help from the experimenter. Children assigned to the control group did not receive training. Instead, they were allowed to play with the experimenter for an equivalent time period of 10 min (the training was estimated to last around 10 min). Therefore, children in the control group did not acquire, through training, relevant knowledge to apply to the test task and *de facto* did not participate in a transfer procedure. Children were also assigned into either a 10-min short delay or a 24-h long delay before being presented with the test task.

The test task included three tools: a stripe-patterned functional tool (dot-patterned in the baseline), a dot-patterned non-functional tool (stripe-patterned in the baseline), and a wave-patterned useless tool (same as in the baseline). Therefore, although the functional features of the tools remained the same, there was now a salient perceptual mismatch between the current and the previously used tools. Children received up to three opportunities to interact with the test task, from picking up a tool, through using it on the apparatus, to abandoning the tool. Independently of whether they solved the tests or not, children received stickers and age-appropriate toys as tokens of appreciation for their participation.

Immediately after three unsuccessful attempts at solving the test task, the perceptually mismatching set of tools was substituted with another set, comprising a functional, non-functional and useless tool, all decorated with a uniform x-pattern (see Figure 1). In this extra testing round, the child was allowed three new attempts at solving the test task with the set of tools.

### Coding and Statistical Analysis

For each video, the child’s score and interactions with the apparatus were coded frame-by-frame in ELAN 4.9.4. An interaction was defined as the time interval between onset and offset of physical contact of a tool held by the participant and a component of the apparatus. The tool could be functional (F), non-functional (NF) or useless (U), and the component of the apparatus could be relevant (rel) or irrelevant (irrel; see Supplementary Datasets 1 and 2). The child could only release the toy-bee by interacting with the relevant components of the apparatus. These components differed between apparatuses (see Figure 1). The following variables were used as the response variables in the analyses:

(a) Score in the test, defined as the outcome of the test, equal to 0 if the child failed to solve the test, and 1 if the child solved the test within the first three attempts. This variable was dichotomous.
(b) Functional tool × relevant components, defined as a proportion of interaction time for the functional tool and the relevant components to the overall interaction time in the test. This variable was continuous. Interaction times for the functional tool and the relevant components followed a right-skewed distribution (Mdn = 0.474, min = 0, max = 1). Residuals of the generalized linear model with this variable as a response were normally distributed.
(c) Functional tool × irrelevant components, defined as a proportion of interaction time for the functional tool and the irrelevant components to the overall interaction time in the test. This variable was continuous. Interaction times for the functional tool and the irrelevant components followed a right-skewed distribution (Mdn = 0, min = 0, max = 1). Residuals of the generalized linear model with this variable as a response variable were not normally distributed, and followed Beta distribution.
(d) Score in the extra testing round, defined as the outcome of the extra test, equal to zero if the child failed to solve the extra test, and 1 if the child solved the extra test within the three attempts received after failing the test. This variable was dichotomous.

Two raters coded 64 and 36% of the videos, respectively. A third, independent rater coded all the material. Time-unit kappa, defined as the overlap between the interval patterns generated by the raters for each recording (Bakeman et al., 2009) was equal to 0.99. For each recording, several variables were computed from the coded interactions (see Supplementary Dataset 1 and 2).

Because of its small size, the control group was not included in the main statistical analyses. However, Fisher’s exact test was run to statistically compare performance between the control and the experimental group. Each hypothesis was addressed with the following statistical tools.

(H1, H2) A generalized linear model, with the Score in the test as the response variable and two predictor variables: Delay (short vs. long) and Age (continuous). The response variable was dichotomous.

(H3, H4, H5) A linear model was used, with the proportion of the interaction time for the functional tool and the relevant components to the overall interaction time as the response variable and three predictor variables: Delay (short vs. long), Age (continuous), and the Score in the test (0 vs. 1). The Shapiro–Wolf test was run to determine the residuals’ distribution. The residuals were normally distributed. Best model selection was performed and the predictors that were not involved in significant main and/or interaction effects were dropped. Furthermore, a generalized linear model was used, with the proportion of the interaction time for the functional tool and the irrelevant components to the overall interaction time as the response variable and three predictor variables: Delay (short vs. long), Age (continuous), and the Score in the test (0 vs. 1). The Shapiro–Wolf test was run to determine the residuals’ distribution. The residuals were not normally distributed, and the variable followed a Beta distribution. Best model selection was performed and the predictors that were not involved in significant main and/or interaction effects were dropped.

(H6) A generalized linear model, with the Score in the test as the response variable and two predictor variables: Delay (short vs. long) and Age (continuous), would be used, but too few children succeeded in the extra test to permit this analysis.

All analyses were conducted in R (v.3.5.1, the R Foundation for Statistical Computing^1^). For hypotheses testing, best model selection for generalized linear models was carried out with the following functions: glm (glmulti package; Calcagno, 2013), dredge, get.models (MuMIn package; Barton, 2020), and Anova (car package; Fox and Weisberg, 2011). Contrasts were calculated with glht function from multcomp package (Hothorn et al., 2008) and power was estimated with Nagelkerke’s R2, using PseudoR2 function from DescTools package Signorell et al., 2019). Weights equal to the total interaction time per child were specified in each model, so that children who did not interact with the box would be excluded from the interactions’ analyses. The results were plotted with the interactions package (Long, 2019). Significance level was set at 0.05.

## Results

All children between 24 and 29 months (*n* = 10) failed to solve the test, and therefore this group was excluded from generalized linear models. Instead, Fisher’s exact test was run to statistically compare performance between children between 24 and 29 months and children older than 29 months. Among children who received training (*n* = 90), 46% (*n* = 41) solved the test, 43% (*n* = 18) of whom after the short delay (for details see Table 1). None of the children who did not receive training (*n* = 15) solved the test, and the probability of doing so was significantly lower than for children who received training (*p* = 0.004). Therefore, training was prerequisite for solving the test. In the short delay group, the delay between baseline and test was much shorter than 10 min as children in the experimental group needed shorter trainings than planned, 2 min on average (*M* = 2.02, SD = 0.87), and children in the control group played shorter with the experimenter than planned, 5.5 min on average (*M* = 5.36, SD = 0.88). Five children who received training (29, 31, 37, 43, and 47 months; compared to two who did not receive training, 32 and 49 months) did not interact with the test task and were excluded from the interactions’ analyses.

**TABLE 1:** An overview of participants by age, outcome, condition, and delay.

	Age	Outcome	Group				Probability of Success After Training
			Without training		With training		
			Short delay	Long delay	Short delay	Long delay	
Test Tools with perceptual mismatch	24–29	Success	0	0	0	0	0%
		Fail	2	1	5	2	
	30–36	Success	0	0	6	4	52.6%
		Fail	2	1	4	5	
	37–43	Success	0	0	8	6	51.9%
		Fail	2	2	5	8	
	44–49	Success	0	0	7	6	43%
		Fail	2	1	10	7	
	50–55	Success	0	0	2	2	57%
		Fail	1	1	1	2	
Extra test Tools without perceptual mismatch	24–29	Success	0	0	0	0	0%
		Fail	0	0	5	2	
	30–36	Success	0	0	2	0	25%
		Fail	0	1	2	4	
	37–43	Success	0	0	0	1	8.3%
		Fail	0	2	5	6	
	44–49	Success	0	0	2	0	11%
		Fail	2	0	9	7	
	50–55	Success	0	0	0	0	0%
		Fail	0	1	1	2	

Among 54 children who failed to solve the test with a perceptually incongruent set of tools and proceeded to the extra test (*N* = 54, 26 boys/28 girls), the children’s ages ranged between 24 and 53 months, mean age was *M* = 40.24 months (SD = 7.76). In line with hypothesis H6, only 9% (*n* = 5) of all children solved the test when the perceptual mismatch within the tool set was removed (see Table 1). Due to the small size of this group, further statistical analyses based on generalized linear modeling were not possible. Only categorical data analysis with Hildebrand’s Del was possible (Hildebrand et al., 1977; Drazin and Kazanjian, 2017) and showed that, after the perceptual mismatch was removed, chances of solving the test were low, but not non-existent.

### H1: Children Are More Likely to Solve the Test Task After Training

In line with our hypothesis, none of the children who did not receive training solved the test. Further, all children younger than 30 months also failed to solve the test, regardless of the delay between the training and the test and were significantly more likely to fail the test than older children (*p* = 0.015). However, even in older children that received the training, the Score did not depend on Age, as the main effect of Age was not significant [χ^2^(1) = 0.561, *p* = 0.454, *R*^2^ = 0.026].

### H2: Compared to Older Children, Younger Children Are Not Less Likely to Solve the Test Task After 24-h Delay

Contrary to our hypothesis, compared to older children, younger children were not less likely to solve the test task after a 24-h delay, as the Age × Delay interaction was not significant [χ^2^(1) = 1.039, *p* = 0.308, *R*^2^ = 0.026].

### H3 and H4: After a Long Delay, Older Children Interact Shorter With the Functional Tool and the Relevant Components

**FIGURE 3:**
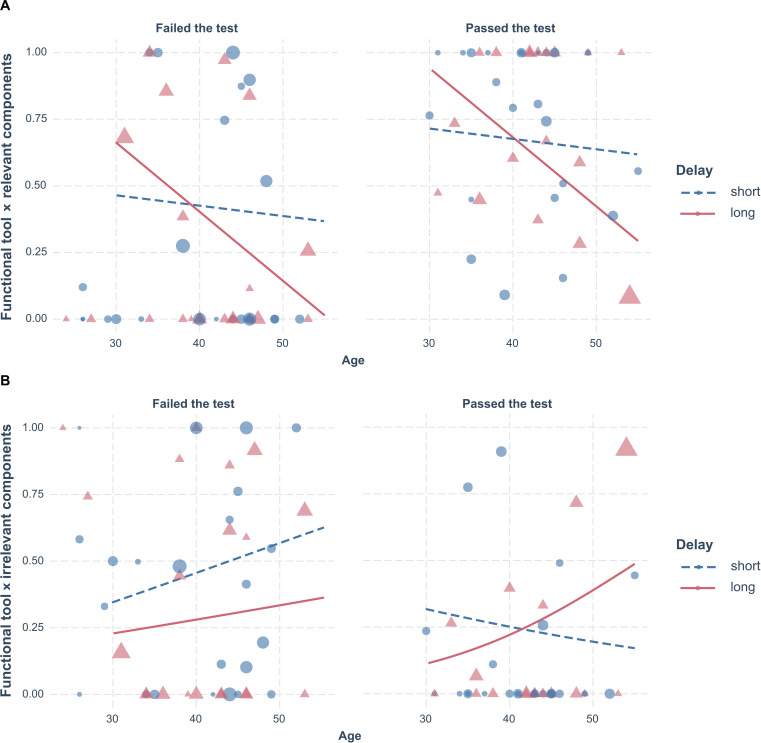
A plot of Age × Delay effect on the proportion of interactions with the functional tool and **(A)** the relevant components, **(B)** the irrelevant components of the apparatus in the test. Note that Age and Delay were involved in a two-way interaction effect in **(A)**, and a three-way interaction effect with the Score in the test in **(B)**. Children younger than 30 months were not involved in the analyses behind the plot, but their results are displayed in the plot for comparison. Circles stand for individual datapoints in the short-delay condition, and triangles stand for individual datapoints in the long-delay condition.

Contrary to hypothesis H3, older children who received training did not interact longer with the functional tool and the relevant components of the apparatuses, as the main effect of Age was not significant [*F*(1,74) = 0.13, *p* = 0.718]. Further, in line with hypothesis H4, there was a significant interaction effect for Age × Delay [*F*(2,74) = 6.385, *p* = 0.003, *R*^2^ = 0.28]. However, the effect was partially different than the predicted one. After the short delay, there was no difference in interacting with the functional tool and the relevant components across ages, but after the long delay, older children engaged in such interactions significantly less than younger children. Note that this effect is opposite to the predicted one (see Figure 3).

A more complex picture emerged from the analysis of interactions with the functional tool and the irrelevant components, since interaction Age × Delay was implicated in a three-way effect for Age × Delay × Success (β = 0.092, SE = 0.001, *z* = 147.51, *p* < 0.001, *R*^2^ = 0.105). Namely, after both delays, with age, children that failed the test interacted more with the functional tool and the irrelevant components (see Figure 3). This was somewhat different for children that solved the test. After the long delay, the older children interacted more with the functional tools and the irrelevant components than the younger children, but after the short delay, this pattern was reversed (see Figure 3).

### H5: Solving the Test Task Involves Longer Interactions With the Functional Tool and Relevant Apparatus Components

In line with our hypothesis, children who solved the test task interacted longer with the functional tool and relevant apparatus components, compared to children who did not, as there was a main effect of Score in the test [*F*(1,74) = 9.674, *p* = 0.003, *R*^2^ = 0.28] and this variable was not implicated in any interactions Further, after the short delay, solving the test involved shorter interactions with the functional tool and the irrelevant components in the older children than in the younger children. After the long delay, however, solving the test involved longer interactions with the functional tool and the irrelevant components in the older than in the younger children (see Figure 3).

## Discussion

In the current study, we tested a novel experimental setup with 2–4 1/2 year-olds to pinpoint the developmental trajectory of flexible tool-dependent problem solving. For the first time, we investigated analogical transfer of actual tool use, both immediately after training and after a 24-h delay. Contrary to previous findings, suggesting that younger children may have difficulty in analogical transfer from long-term memory, we found that once children pass the 30-month threshold, they can transfer solutions that depend on prioritizing tool function over appearance across problems, regardless of the delay between the problems. Contrary to previous studies with so young children, language demands of the task were kept to a minimum, as children received puzzle boxes instead of stories and tackled the test without any verbal or non-verbal guidance from the experimenter.

### Analogical Transfer Was Not Possible Before the Age of 2.5 Years

Children younger than 2 1/2 years did not manage to solve the problem, even if they learnt the correct solution on a perceptually dissimilar problem ten min earlier. However, this was not the case for the children between 2 1/2 and 4 1/2, who, after learning the correct solution 10 min or 24 h earlier, could solve the problem regardless of the delay. Therefore, it seems that neither the delay nor the perceptual mismatch within the tool set impeded the transfer at these ages. Given previous findings from analogical transfer studies (Crisafi and Brown, 1986), it is not surprising that children younger than 2 1/2 years did not transfer the solution between two physically dissimilar problems, but it is surprising that ability to transfer was independent of age in children between 30 and 55 months of age.

The current analogical transfer task perhaps required simpler analogical reasoning that other classical tasks, e.g., of the A:B:C:D type (Thibaut and French, 2016). Typically, such tasks require detecting how A is related to B (e.g., A fits in B, the shirt fits in the suitcase) and then applying this relation to a pair of C and D items (e.g., C fits in D, the toy car fits in the box). This is a complex task that involves holding “online” and transferring an abstract rule, an operation that lies at the core of adult analogical reasoning. However, from the child’ point of view, the task is fairly abstract, requires well-developed verbal skills and does not allow any agency on the child’s part. On the contrary, in the current task, children needed only to hold and transfer a concrete rule across one pair of items (A can be solved as B), use no language and act as agents. This may explain why children’s analogical transfer was independent of age in children between 30 and 55 months of age. In the future, our task could be, however, used as an A:B:C:D task, as we devised several pairs of boxes, all relying on the same rule: that both boxes in the pair can be solved with the same tool.

Transferring a solution across problems arguably requires the ability to mentally represent objects and actions involved in the solution. To transfer the solution across two problems, children need to simultaneously consider the source problem, familiar but currently absent, and the target problem, currently present but unfamiliar. In other words, the child needs to activate a mental representation of the source problem in the service of problem solving, which, according to neo-Piagetian accounts may be available in 2-year-olds at the earliest (e.g., Morra et al., 2008). While our findings could be taken to indicate that representational ability is still immature before the 30th month of life, hindering analogical transfer, 2-year olds have been shown to succeed in transfer tasks as long as the similarity between the problems is explicitly stated by the experimenter (Crisafi and Brown, 1986; Goswami, 1991), suggesting at least some capacity to mentally represent the source problem.

That children below 30 months have difficulties in transferring across conceptually similar problems has been shown before also in other tasks, using deferred imitation (Herbert and Hayne, 2000) and object search (DeLoache et al., 2004). Although analogical transfer may be available even for 2-year-olds, they may require the experimenter to highlight the conceptual similarity between the source and the target problem. For instance, Hayne and Gross (2015) showed that 2-year-olds can transfer a sequence of actions across perceptually dissimilar tasks, as long as the experimenter provided the same verbal label to highlight the underlying functional similarity. Afterward, children were also shown to map this similarity onto another set of problems, but only as long as the functional similarity was highlighted with the verbal label within the initial set.

Therefore, in principle, 2-year-olds are able to transfer knowledge across functionally similar contexts but may be less likely to spontaneously notice the link between the source and the target than 2 1/2-year-olds. The challenges posed by the target problem can be also viewed from the sensorimotor perspective, as it demands activating and coordinating several sensorimotor schemes regarding essential features of the problem (the puzzle box, its relevant components, the toy bee inside, the tools), the goal of the problem (selecting a tool and retrieving the toy bee) and the strategy of arriving at this goal (applying the tool to the components of the box; e.g., Morra and Panesi, 2017). Such activation and coordination may limit children’s spontaneous transfer below 30 months.

Alternatively, our youngest participants could have failed to prioritize attending to relevant over irrelevant aspects of the target problem. Between 2 and 2 1/2, children’s attention undergoes a transition, as it becomes increasingly governed by top-down (executive functions), not bottom-up influences (attractiveness and novelty of stimuli; Ruff and Capozzoli, 2003). Then, the participants in this age range would focus to a greater extent than older children on the irrelevant aspects of the problem. This was not the case, however, as the youngest children interacted with the functional tool and the irrelevant aspects to a similar extent as the older children who failed to solve the test.

In fact, it has recently been shown that the capacity to attend to relevant, rather than irrelevant, information in children around the age of 3 years can be substantially boosted by jointly attending to the problem tasks (Psouni et al., 2019). Future studies should address whether the experimenter jointly attending to the task with the youngest children or providing verbal cues that highlight the similarity between the source and the target might boost the youngest participants’ capacity to analogical transfer.

Allowing children to manipulate the tools in future studies, even in the youngest group, may further illuminate whether children’s motor programs for tools change with age. Since tools in the current study were made-up and their function was the same for the source and the target, it is unlikely that previous motor programs for familiar tools, e.g., a spoon, impacted on children’s tool-use flexibility (Barrett et al., 2007). In the future, however, our set-up could be used to study how robust tool-dependent transfer is when the function and motor programs associated with the tools is manipulated across tasks.

### The Interplay of Age and Memory

Our findings suggest that once children are able to transfer, they can do so also with a delay between initial problem and test problem, beyond the 15-min limit identified by Scarf and colleagues for 3-year-olds in another task (Scarf et al., 2013). Children could not simply repeat the previously learnt motor action in order to succeed, as the training and test puzzles were distinctively different in that sense; instead, they needed to generalize the action to a different looking problem. Thus, it is unlikely that children could solve the problem relying on procedural memory (note also that procedural learning does not generalize well, see, e.g., Doll et al., 2015).

Interestingly, our result mirrors the findings from Herbert and Hayne’s deferred imitation study with 30-month-olds, where children’s transfer was immune to a similar 24-h-delay (2005). In another above-mentioned study, this performance was also achievable for 2-year-olds as long as their attention was drawn to the functional similarity between two perceptually dissimilar tasks, suggesting that 2-year-olds’ working memory resources may suffice for analogical transfer (Herbert and Hayne, 2000). Assuming that working memory is not separate from long-term memory, but rather a state of activation encompassing certain information stored in long-term memory (Oberauer, 2002; Pascual-Leone and Johnson, 2005), it is possible that once analogical transfer is permitted by working memory maturity, it works equally well for the immediate and delayed problems, at least by 24 h.

In terms of outcome, children performed similarly regardless of the delay and age in our analogical transfer task, that is, children were equally likely to solve the test after 10 min and 24 h. However, the analysis of the interaction patterns reveals a different picture. In the short-term, attending to the relevant components of the problem was similar in children of different ages, and lower in those that did not solve the task than those that did. In the long term, however, among children above 30 months, older children attended shorter to the relevant components of the problem than the younger children. Note that this was true both for the children that failed and those that passed the test. Taken together, these results suggest that, even if children reach the same outcome in the short and in the long term, retrieval from long-term memory does not pose a uniform challenge in children of different ages.

We posit that it is highly unlikely that retrieving a relevant experience from long-term memory is increasingly difficult as child’s memory matures. Instead, we suggest that in the long term, older children may adopt a more flexible, explorative approach than younger children, spending more time on interactions with the irrelevant aspects of the target problem. Perhaps with age, the solution to the target problem becomes increasingly straightforward and children seek additional ways of solving it before acting on the relevant aspects of the problem.

This reasoning seems to be supported by the analysis of interactions with the functional tool and the irrelevant components. Among children that passed the test, the older children interacted more with the functional tool and the irrelevant components than the younger ones, but only in the long term. This pattern was different among children that failed the test, since there was no difference in interactions with the functional tool and the irrelevant components across ages, both in the short and in the long term.

Repeating the previously learnt motor action clearly did not suffice for successful transfer, as focusing on the functional tool without focusing on the relevant components did not differ between children that succeeded and those that did not, at least in the short-term. In other words, it was not sufficient for the child to pick up the functional tool and apply it to different components; rather, the child had to understand which components matched the tool’s function. Further, children had to transfer the solution acquired in a specific one-time personal episode and flexibly apply it to the novel situation, which requires episodic memory (Tulving, 2005). Therefore, the present results suggest that the ability for non-verbal transfer across physically dissimilar problems in young children has been underestimated. It seems that between 2 1/2 and 4 1/2 years of age children can perform such transfers, using episodic memory, as long as the success does not require the comprehension of verbal instructions.

### Limitations

The manipulations introduced in the current experiment could not disentangle between two possible reasons behind children’s failures: a difficulty caused by analogical transfer or by the perceptual mismatch within the tool set. When the perceptual mismatch was removed, very few children improved their performance, suggesting that the failures were predominantly caused by the difficulty in analogical transfer. However, as this extra test involved the same children and followed immediately after the test, failure to solve the task could also be due to a drop in children’s motivation after failure, interference between the perceptually incongruent and the uniform tool sets, or fatigue due to prolonged testing. Future studies ought to disentangle between these possible explanations through, for instance, testing one group of children with the perceptually mismatching tools, and another group with the same tools as in the training.

In the present study, group sizes for the youngest and oldest children were small (*n* = 10 and *n* = 9, respectively). Since it is possible that the youngest children were motorically disadvantaged compared to older children, future studies might include a preferential looking test for the youngest children. Alternatively, to limit the motor involvement on the child’s part but retain the current setup, the experimenter could use tools indicated by the child, as in Pauen and Bechtel-Kuehne’s study (2016). However, the present experimental setup allowed executing multiple motor actions with the chosen tool, enhancing the child’s active involvement and independence while maintaining a minimal language task demand. Having the experimenter handle the tools as instructed by the child would significantly increase language task demands, thereby hindering a comparison of performance across age groups and children with varying language abilities.

The current design could not disentangle whether children attended to the perceptual differences between tool handles and disregarded them in favor of the tools’ functionality, or whether they did not attend to the tools’ appearance at all. Drawing the children’s attention to the tools’ appearance would, again, have resulted in increased language demands of the task. Future investigations may opt to in retrospect ask the children about the tools’ appearance, in an effort to disentangle these two possibilities.

## Conclusion

We tested a novel set-up to investigate analogical transfer of tool use in 2–4 1/2-year-olds and showed that transfer between functionally similar, but perceptually dissimilar problems can be as robust after 10 m to a 24-h delay. Children below 30 months did not demonstrate such transfer, in line with previous studies that, like ours, limited verbal cues pointing to the similarity between the source and the target task. Interestingly, we found that, even when children’s behavior in the test led to the same outcome regardless of age and delay, this behavior had different trajectories. Therefore, we posit that future studies should focus not only on the outcome of children’s actions but also on behavioral patterns that lead to those outcomes.

The ability of flexible tool-dependent problem solving has a remarkable impact on everyday life and decision making, both in the local and the global context (Keen, 2011). Recognizing the principles for solution across physical and abstract problems allows for efficient and timely action in response to grave and actual challenges, such as climate change (Keen, 2011). Understanding how children spontaneously shift attention toward relevant aspects of solutions and problems could inform future interventions, on the one hand, enhancing efficient problem solving from a young age, and on the other, enhancing spontaneous focusing on relevant aspects of abstract problems in adults. Furthermore, as analogical transfer of tool use in the current setup did not require verbal instructions, the pairs of problems and tools could be tested with clinical populations of children and adults with speech and/or hearing impediments or impairments.

## Data Availability Statement

All datasets generated in this study are included in the article/Supplementary Material.

## Ethics Statement

The studies involving human participants were reviewed and approved by Swedish Ethical Review Authority in Lund (DRN 2018/572, PI Psouni). Written informed consent to participate in this study was provided by the participants’ legal guardian/next of kin.

## Author Contributions

KB: conceptualization, methodology, data curation, formal analysis, writing – original draft, visualization, and funding acquisition. FL and ML: methodology, investigation, and data curation. EP: methodology, writing – review and editing, supervision, project administration, and funding acquisition. All authors contributed to the article and approved the submitted version.

## Conflict of Interest

The authors declare that the research was conducted in the absence of any commercial or financial relationships that could be construed as a potential conflict of interest.

## Footnotes

1 http://www.R-project.org
