# Supplementary material for: Flexibility in Problem Solving: Analogical Transfer of Tool Use in Toddlers Is Immune to Delay
Source: Front Psychol. 2020 Oct 6;11:573730. doi: 10.3389/fpsyg.2020.573730 (PMC7573132; doi:10.3389/fpsyg.2020.573730)

Supplementary Information 1

# Supplementary Data

**Supplementary Dataset 1.** A dataset with Participant ID, Condition (experimental vs. control), Delay (short vs. long), Task set (A – G) and Success (pass vs. fail) in Experiment 1.

| Participant ID | Condition | Delay | Task set | Success |
| --- | --- | --- | --- | --- |
| ID1 | experimental | long | A | 1 |
| ID2 | experimental | short | D | 0 |
| ID3 | experimental | long | A | 0 |
| ID4 | experimental | short | D | 1 |
| ID5 | experimental | long | A | 1 |
| ID6 | experimental | long | D | 0 |
| ID7 | experimental | short | D | 1 |
| ID8 | experimental | long | F | 0 |
| ID9 | experimental | long | B | 0 |
| ID10 | experimental | short | D | 1 |
| ID11 | experimental | short | A | 0 |
| ID12 | experimental | long | A | 0 |
| ID13 | experimental | long | A | 0 |
| ID14 | experimental | short | B | 0 |
| ID15 | experimental | long | D | 1 |
| ID16 | experimental | long | D | 1 |
| ID17 | experimental | long | D | 1 |
| ID18 | experimental | short | A | 1 |
| ID19 | experimental | short | D | 0 |
| ID20 | experimental | short | D | 0 |
| ID21 | experimental | long | C | 0 |
| ID22 | experimental | short | A | 1 |
| ID23 | experimental | long | A | 1 |
| ID24 | experimental | long | B | 0 |
| ID25 | experimental | short | B | 1 |
| ID26 | experimental | long | A | 0 |
| ID27 | experimental | short | D | 1 |
| ID28 | experimental | short | D | 1 |
| ID29 | experimental | short | D | 0 |
| ID30 | experimental | long | A | 0 |
| ID31 | experimental | short | A | 1 |
| ID32 | experimental | short | D | 1 |
| ID33 | experimental | long | D | 1 |
| ID34 | experimental | short | D | 1 |
| ID35 | experimental | long | D | 0 |
| ID36 | experimental | long | A | 0 |
| ID37 | experimental | long | D | 1 |
| ID38 | experimental | long | D | 1 |
| ID39 | experimental | short | D | 0 |
| ID40 | experimental | short | D | 0 |
| ID41 | experimental | long | A | 1 |
| ID42 | experimental | long | A | 1 |
| ID43 | experimental | short | D | 0 |
| ID44 | experimental | short | A | 1 |
| ID45 | experimental | long | D | 1 |
| ID46 | experimental | long | A | 1 |
| ID47 | experimental | short | B | 0 |
| ID48 | experimental | long | A | 0 |
| ID49 | experimental | long | A | 1 |
| ID50 | experimental | short | A | 0 |
| ID51 | experimental | short | A | 0 |
| ID52 | experimental | short | A | 0 |
| ID53 | experimental | short | A | 1 |
| ID54 | experimental | long | D | 0 |
| ID55 | experimental | long | A | 1 |
| ID56 | experimental | short | A | 0 |
| ID57 | experimental | short | D | 0 |
| ID58 | experimental | short | C | 1 |
| ID59 | experimental | long | A | 0 |
| ID60 | experimental | long | D | 1 |
| ID61 | experimental | short | D | 0 |
| ID62 | experimental | long | D | 0 |
| ID63 | experimental | long | A | 1 |
| ID64 | experimental | short | A | 0 |
| ID65 | experimental | long | B | 0 |
| ID66 | experimental | long | A | 0 |
| ID67 | experimental | short | A | 1 |
| ID68 | experimental | long | F | 1 |
| ID69 | experimental | short | A | 0 |
| ID70 | experimental | short | A | 0 |
| ID71 | experimental | short | A | 0 |
| ID72 | experimental | short | D | 1 |
| ID73 | experimental | short | D | 1 |
| ID74 | experimental | long | F | 0 |
| ID75 | experimental | long | B | 0 |
| ID76 | experimental | long | A | 0 |
| ID77 | experimental | short | A | 1 |
| ID78 | experimental | short | F | 0 |
| ID79 | experimental | short | A | 0 |
| ID80 | experimental | short | A | 0 |
| ID81 | experimental | long | A | 0 |
| ID82 | experimental | short | D | 1 |
| ID83 | experimental | short | D | 1 |
| ID84 | experimental | short | A | 1 |
| ID85 | experimental | long | A | 0 |
| ID86 | experimental | short | B | 0 |
| ID87 | experimental | long | D | 0 |
| ID88 | experimental | short | A | 0 |
| ID89 | experimental | short | G | 1 |
| ID90 | experimental | short | D | 1 |
| ID91 | control | short | F | 0 |
| ID92 | control | short | D | 0 |
| ID93 | control | long | D | 0 |
| ID94 | control | short | F | 0 |
| ID95 | control | long | A | 0 |
| ID96 | control | short | D | 0 |
| ID97 | control | short | A | 0 |
| ID98 | control | long | D | 0 |
| ID99 | control | short | B | 0 |
| ID100 | control | long | F | 0 |
| ID101 | control | long | D | 0 |
| ID102 | control | long | F | 0 |
| ID103 | control | short | A | 0 |
| ID104 | control | short | A | 0 |
| ID105 | control | short | A | 0 |

I

**Supplementary Dataset 2.** A dataset with Participant ID and interaction times [msec]. For details of the variables please see Table S1.

| Participant ID | Overall interaction time | F_rel_total_raw | F_irrel_total_raw | NF_rel_total_raw | NF_irrel_total_raw | U_rel_total_raw | U_irrel_total_raw |
| --- | --- | --- | --- | --- | --- | --- | --- |
| ID1 | 9190 | 9190 | 0 | 0 | 0 | 0 | 0 |
| ID2 | 0 | 0 | 0 | 0 | 0 | 0 | 0 |
| ID3 | 8180 | 0 | 8180 | 0 | 0 | 0 | 0 |
| ID4 | 7880 | 1220 | 0 | 4640 | 2020 | 0 | 0 |
| ID5 | 5930 | 5930 | 0 | 0 | 0 | 0 | 0 |
| ID6 | 1630 | 0 | 1630 | 0 | 0 | 0 | 0 |
| ID7 | 7330 | 6520 | 810 | 0 | 0 | 0 | 0 |
| ID8 | 20120 | 0 | 0 | 0 | 15900 | 0 | 4220 |
| ID9 | 4925 | 0 | 4345 | 0 | 580 | 0 | 0 |
| ID10 | 1130 | 1130 | 0 | 0 | 0 | 0 | 0 |
| ID11 | 10870 | 8110 | 1230 | 0 | 1530 | 0 | 0 |
| ID12 | 20880 | 17850 | 0 | 3030 | 0 | 0 | 0 |
| ID13 | 17150 | 14380 | 0 | 0 | 2770 | 0 | 0 |
| ID14 | 11460 | 0 | 8730 | 0 | 1490 | 0 | 1240 |
| ID15 | 3010 | 3010 | 0 | 0 | 0 | 0 | 0 |
| ID16 | 9020 | 3360 | 0 | 0 | 0 | 0 | 5660 |
| ID17 | 12340 | 12340 | 0 | 0 | 0 | 0 | 0 |
| ID18 | 7620 | 3470 | 0 | 3000 | 0 | 1150 | 0 |
| ID19 | 1120 | 0 | 0 | 0 | 1120 | 0 | 0 |
| ID20 | 860 | 0 | 860 | 0 | 0 | 0 | 0 |
| ID21 | 24630 | 0 | 22550 | 0 | 0 | 0 | 2080 |
| ID22 | 12760 | 12760 | 0 | 0 | 0 | 0 | 0 |
| ID23 | 13400 | 7880 | 0 | 630 | 0 | 4890 | 0 |
| ID24 | 0 | 0 | 0 | 0 | 0 | 0 | 0 |
| ID25 | 1750 | 1750 | 0 | 0 | 0 | 0 | 0 |
| ID26 | 12406 | 4770 | 5516 | 0 | 0 | 0 | 2120 |
| ID27 | 1880 | 1880 | 0 | 0 | 0 | 0 | 0 |
| ID28 | 2620 | 2620 | 0 | 0 | 0 | 0 | 0 |
| ID29 | 7330 | 7330 | 0 | 0 | 0 | 0 | 0 |
| ID30 | 26670 | 6830 | 18360 | 0 | 1480 | 0 | 0 |
| ID31 | 12340 | 2780 | 9560 | 0 | 0 | 0 | 0 |
| ID32 | 5420 | 5420 | 0 | 0 | 0 | 0 | 0 |
| ID33 | 9270 | 9270 | 0 | 0 | 0 | 0 | 0 |
| ID34 | 2070 | 930 | 0 | 820 | 0 | 320 | 0 |
| ID35 | 1660 | 0 | 0 | 0 | 0 | 1660 | 0 |
| ID36 | 9410 | 0 | 0 | 0 | 0 | 0 | 9410 |
| ID37 | 6560 | 6560 | 0 | 0 | 0 | 0 | 0 |
| ID38 | 7460 | 7460 | 0 | 0 | 0 | 0 | 0 |
| ID39 | 1180 | 0 | 0 | 0 | 1180 | 0 | 0 |
| ID40 | 2110 | 0 | 1050 | 0 | 500 | 0 | 560 |
| ID41 | 7900 | 5280 | 2620 | 0 | 0 | 0 | 0 |
| ID42 | 19230 | 19230 | 0 | 0 | 0 | 0 | 0 |
| ID43 | 11870 | 11870 | 0 | 0 | 0 | 0 | 0 |
| ID44 | 16740 | 1530 | 15210 | 0 | 0 | 0 | 0 |
| ID45 | 2480 | 2480 | 0 | 0 | 0 | 0 | 0 |
| ID46 | 9920 | 7290 | 2630 | 0 | 0 | 0 | 0 |
| ID47 | 31400 | 0 | 31400 | 0 | 0 | 0 | 0 |
| ID48 | 15660 | 15660 | 0 | 0 | 0 | 0 | 0 |
| ID49 | 10480 | 6330 | 4150 | 0 | 0 | 0 | 0 |
| ID50 | 10580 | 0 | 10580 | 0 | 0 | 0 | 0 |
| ID51 | 36600 | 36600 | 0 | 0 | 0 | 0 | 0 |
| ID52 | 0 | 0 | 0 | 0 | 0 | 0 | 0 |
| ID53 | 9140 | 7380 | 0 | 1750 | 0 | 0 | 10 |
| ID54 | 0 | 0 | 0 | 0 | 0 | 0 | 0 |
| ID55 | 19020 | 8520 | 1260 | 2490 | 6750 | 0 | 0 |
| ID56 | 7020 | 0 | 4600 | 0 | 2420 | 0 | 0 |
| ID57 | 7850 | 0 | 0 | 0 | 0 | 0 | 7850 |
| ID58 | 10287 | 10287 | 0 | 0 | 0 | 0 | 0 |
| ID59 | 2970 | 340 | 1750 | 0 | 460 | 0 | 420 |
| ID60 | 3800 | 1800 | 0 | 2000 | 0 | 0 | 0 |
| ID61 | 5480 | 0 | 1810 | 0 | 1150 | 0 | 2520 |
| ID62 | 4770 | 0 | 0 | 0 | 1470 | 0 | 3300 |
| ID63 | 17290 | 4900 | 12390 | 0 | 0 | 0 | 0 |
| ID64 | 27870 | 0 | 27870 | 0 | 0 | 0 | 0 |
| ID65 | 7880 | 0 | 0 | 1200 | 0 | 4530 | 2150 |
| ID66 | 38750 | 26390 | 6060 | 1460 | 350 | 0 | 4490 |
| ID67 | 6260 | 3480 | 2780 | 0 | 0 | 0 | 0 |
| ID68 | 60410 | 5000 | 55410 | 0 | 0 | 0 | 0 |
| ID69 | 14010 | 0 | 7005 | 0 | 7005 | 0 | 0 |
| ID70 | 7580 | 910 | 4410 | 770 | 0 | 0 | 1490 |
| ID71 | 25750 | 13340 | 5010 | 3260 | 2480 | 0 | 1660 |
| ID72 | 2040 | 2040 | 0 | 0 | 0 | 0 | 0 |
| ID73 | 12140 | 12140 | 0 | 0 | 0 | 0 | 0 |
| ID74 | 5780 | 0 | 4290 | 0 | 0 | 0 | 1490 |
| ID75 | 19136 | 0 | 11771 | 0 | 990 | 0 | 6375 |
| ID76 | 5130 | 0 | 0 | 0 | 4710 | 0 | 420 |
| ID77 | 14500 | 5630 | 0 | 580 | 8290 | 0 | 0 |
| ID78 | 9820 | 0 | 4060 | 0 | 4310 | 0 | 1450 |
| ID79 | 26830 | 24090 | 2740 | 0 | 0 | 0 | 0 |
| ID80 | 10206 | 0 | 5586 | 0 | 3340 | 0 | 1280 |
| ID81 | 6600 | 0 | 5670 | 0 | 930 | 0 | 0 |
| ID82 | 2990 | 2990 | 0 | 0 | 0 | 0 | 0 |
| ID83 | 5750 | 2930 | 2820 | 0 | 0 | 0 | 0 |
| ID84 | 16880 | 12540 | 4340 | 0 | 0 | 0 | 0 |
| ID85 | 14905 | 15175 | 0 | 430 | 0 | 0 | 0 |
| ID86 | 37150 | 10210 | 17850 | 0 | 9090 | 0 | 0 |
| ID87 | 0 | 0 | 0 | 0 | 0 | 0 | 0 |
| ID88 | 5150 | 4500 | 0 | 650 | 0 | 0 | 0 |
| ID89 | 7430 | 5680 | 1750 | 0 | 0 | 0 | 0 |
| ID90 | 7350 | 5830 | 0 | 0 | 580 | 940 | 0 |
| ID91 | 6370 | 0 | 0 | 6370 | 0 | 0 | 0 |
| ID92 | 0 | 0 | 0 | 0 | 0 | 0 | 0 |
| ID93 | 108360 | 108360 | 0 | 0 | 0 | 0 | 0 |
| ID94 | 11740 | 0 | 980 | 0 | 7360 | 0 | 3400 |
| ID95 | 116048 | 116048 | 0 | 0 | 0 | 0 | 0 |
| ID96 | 0 | 0 | 0 | 0 | 0 | 0 | 0 |
| ID97 | 29204 | 0 | 14602 | 0 | 14602 | 0 | 0 |
| ID98 | 15940 | 0 | 0 | 0 | 15940 | 0 | 0 |
| ID99 | 44260 | 0 | 0 | 0 | 44260 | 0 | 0 |
| ID100 | 12660 | 12660 | 0 | 0 | 0 | 0 | 0 |
| ID101 | 16300 | 14800 | 1500 | 0 | 0 | 0 | 0 |
| ID102 | 12640 | 0 | 8790 | 0 | 2710 | 0 | 1140 |
| ID103 | 10000 | 0 | 10000 | 0 | 0 | 0 | 0 |
| ID104 | 14460 | 1280 | 4185 | 0 | 8995 | 0 | 0 |
| ID105 | 21090 | 8190 | 12900 | 0 | 0 | 0 | 0 |

# Supplementary Figures and Tables

## Supplementary Figures

**Figure S1.** **An overview of a correct solution of (A) the training task, and (B) the test task, both belonging to the B set.**


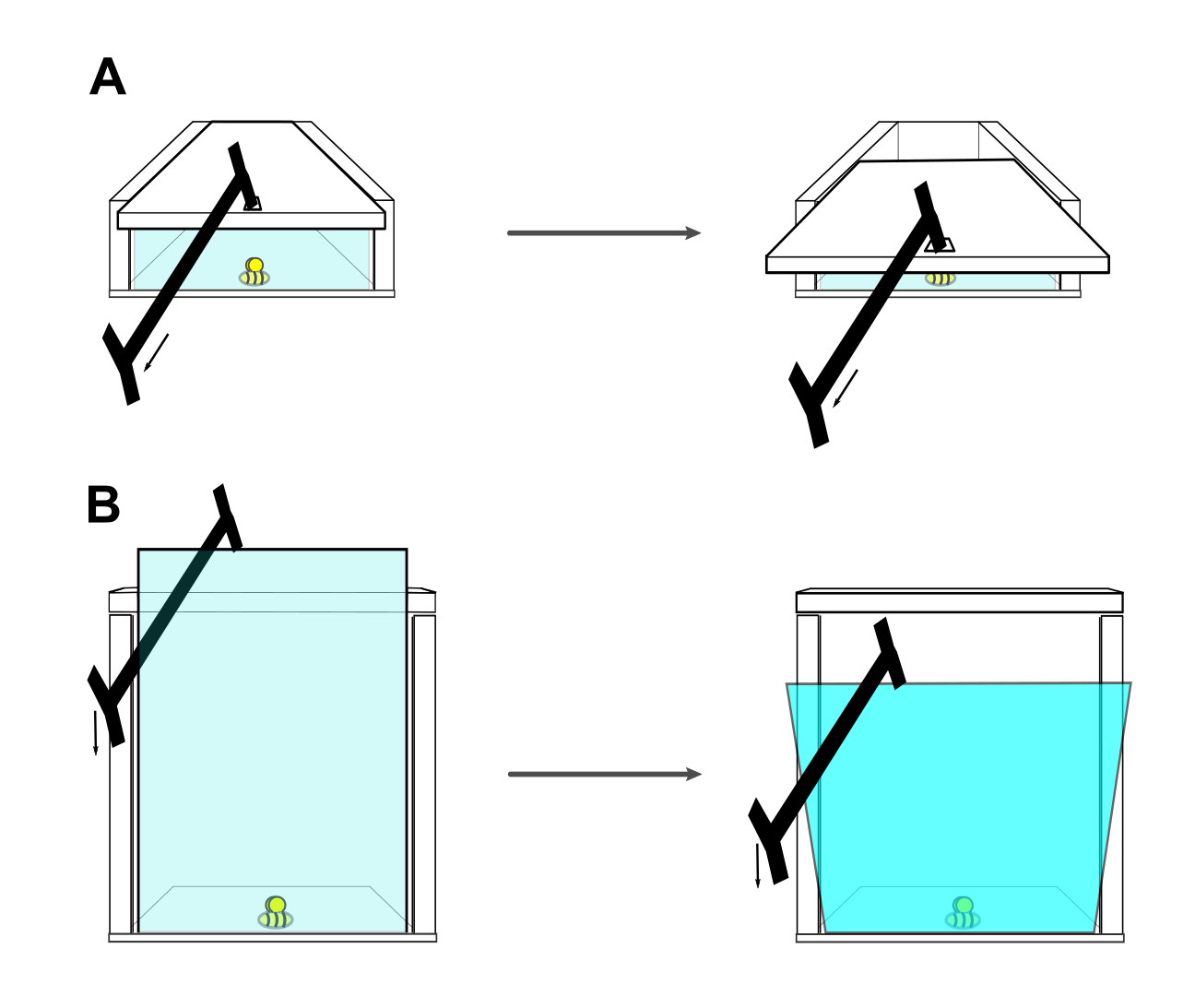

Supplement: Supplementary file 1 [file Data_Sheet_1.docx]
